# *bla*_CTX-M-27_–Encoding *Escherichia coli* Sequence Type 131 Lineage C1-M27 Clone in Clinical Isolates, Germany

**DOI:** 10.3201/eid2310.170938

**Published:** 2017-10

**Authors:** Hiren Ghosh, Swapnil Doijad, Linda Falgenhauer, Moritz Fritzenwanker, Can Imirzalioglu, Trinad Chakraborty

**Affiliations:** Justus Liebig University, Giessen, Germany

**Keywords:** Escherichia coli, ST131, CTX-M-27, ESBL, extended-spectrum β-lactamases, C1-M27, bacteria, Germany, Japan, antimicrobial resistance, *bla*_CTX-M-27_

## Abstract

We examined extended-spectrum β-lactamase–producing isolates from livestock, humans, companion animals, food, and the environment during 2009–2016 in Germany for the presence of CTX-M-27 allele within *Escherichia coli* sequence type (ST) 131. *E. coli* ST131 C1-M27 was exclusively present in humans; its incidence increased from 0% in 2009 to 45% in 2016.

During the past 20 years, *Escherichia coli* sequence type (ST) 131 has emerged as a prevalent vehicle for extended-spectrum β-lactamases (ESBL) worldwide. Particularly prevalent are isolates of the clade ST131 C/H30R, which frequently are associated with urinary tract infections and bacteremia (*1*,*2*). Although the ESBL production of the predominant subgroup ST131 C2/H30Rx is conferred by the CTX-M-15 allele, the emerging subgroup C1 often is associated with other CTX-M alleles, such as CTX-M-14 and CTX-M-27 (*3*). An increase in C1/H30R ST131 isolates was initially reported among clinical isolates in Japan; most of those were identified as members of the recently defined clade C1-M27 (90% of C1/H30R) (*3*). More recently, a dramatic rise from 0% to 65% in the incidence of ST131 C1/H30R *bla*_CTX-M-27_ isolates in the fecal carriage of children in France during 2010–2015 was reported (*4*). In addition, ST131 isolates harboring *bla*_CTX-M-27_ have been reported sporadically from other countries (*3*). We examined ESBL-producing isolates from livestock, humans, companion animals, food, and the environment during 2009–2016 in Germany for the CTX-M-27 allele.

We analyzed a representative subset of 953 sequenced isolates from a collection of 4,386 nonrepetitive ESBL-producing *E. coli*, which were obtained through 2 national research networks investigating the incidence of antimicrobial resistance: ESBL and Fluoroquinolone Resistance in Enterobacteriaceae (RESET) and German Center for Infection Research (DZIF) in Germany (Technical Appendix 1). In silico multilocus sequence typing (MLST) identified 159 (17%) of the 953 isolates as ST131 (Technical Appendix 2). The most prevalent ESBL genes in the studied isolates were *bla*_CTX-M-15_ (73 [46%]), followed by *bla*_CTX-M-27_ (24 [15%]), *bla*_CTX-M-1_ (18 [11%]), *bla*_CTX-M-14_ (15 [9%]), and others (*bla*_CTX-M-3/11/17/24/36/47_) (10 [6%]).

Because recent reports have documented an increase in the number of C1-M27 clade isolates in Japan and France, we investigated *bla*_CTX-M-27_–encoding ST131 isolates in more detail. All ST131 isolates with *bla*_CTX-M-27_ were of serogroup O25b and harbored a *fimH*30 allele, except for 1 isolate that was of serogroup O16 and carried a *fim*H41 allele. Recently, the M27PP1 prophage-like region was defined as a specific marker for C1-M27 clade (*3*,*4*). This region was present in 23 of 24 *bla*_CTX-M-27_–harboring isolates. Phylogenomic analysis revealed that these 23 isolates belong to clade C1/H30R (Technical Appendix 2 Figure).

We identified contigs with F1:A2:B20 plasmid replicons in sequences from 20 of 24 isolates; the remaining isolates harbored contigs with F1:A6:B20, F1:A2:B20, F1:A2:B-, and F29:A-:B10 plasmid incompatibility groups. We sequenced the genome of 1 representative isolate (H105) to completion (GenBank accession numbers: chromosome, CP021454; plasmid, CP021871) and confirmed that the *bla*_CTX-M-27_–encoding contig was indeed part of a plasmid harboring the F1:A2:B20 replicon (*5*). This plasmid is highly conserved in isolates of ST131 and is probably ancestral to the C1/H30R clade because it is present in all the *bla*_CTX-M-27_–positive ST131 isolates, regardless of whether they harbor antimicrobial resistance genes (*5*).

Core genome phylogenetic comparisons of all C/H30R ST131 isolates, based on alignment to the closed genome of *E. coli* ST131 lineage C1-M27 isolate H105, revealed an average of 292 single-nucleotide polymorphisms (SNPs). In contrast, isolates within the C1-M27 clade were separated by <100 SNPs. Comparative analyses of 13 isolates reported from Japan showed that these isolates share ≈85% of the genome with those from Germany. Isolates from both countries exhibit an average difference of 59 SNPs, indicating clonality and possible evolution from a single common ancestor (Technical Appendix 2). Metadata of the C1-M27 isolates showed 19 of 24 isolates were obtained in 2015 and 2016, indicating recent emergence.

Our results provide evidence for the recent emergence of ST131 subgroup *fim*H30-O25b, clade C1-M27, harboring *bla*_CTX-M-27_, in Germany and reinforce observations made elsewhere. The data suggest an ongoing shift in CTX-M alleles associated with ST131 infections worldwide that now warrants further attention.

Technical Appendix 1 Resistance genes from a representative subset of 953 sequenced isolates from a collection of 4,386 nonrepetitive extended-spectrum β-lactamase–producing *Escherichia coli*, Germany.

Technical Appendix 2 Whole-genome sequencing and in silico analysis of *bla*_CTX-M-27_–encoding *Escherichia coli*, Germany.
