# Supplementary material for: blaCTX-M-27–Encoding Escherichia coli Sequence Type 131 Lineage C1-M27 Clone in Clinical Isolates, Germany
Source: Emerg Infect Dis. 2017 Oct;23(10):1754–6. doi: 10.3201/eid2310.170938 (PMC5621564; doi:10.3201/eid2310.170938)
Supplement: Technical Appendix 2 — Whole-genome sequencing and in silico analysis of blaCTX-M-27–encoding Escherichia coli, Germany. [file 17-0938-Techapp-s2.pdf]

Article DOI: https://doi.org/10.3201/eid2310.170938

bla$_{CTX-M-27}$-Encoding *Escherichia coli* Sequence Type 131 Lineage C1-M27 Clone in Clinical Isolates, Germany

Technical Appendix

Whole-Genome Sequencing

On the isolates examined in this study (Technical Appendix Table), we conducted whole-genome sequencing using Illumina platforms (either MiSeq or NextSeq), as described previously (*1*,*2*). Briefly, genomic DNA was isolated from overnight cultures by using the Purelink Genomic DNA Mini kit (Invitrogen, Darmstadt, Germany). For short-read whole-genome sequencing, an Illumina Nextera XT library (Illumina Netherlands BV, Eindhoven, the Netherlands) was constructed and sequenced. The *bla*$_{CTX-M-27}$-encoding *Escherichia coli* sequence type (ST) 131 isolate H105 a member of the lineage C1/H30R was sequenced for its complete genome using PacBio RSII system (Pacific Biosciences, Menlo Park, CA, USA). GenBank accession numbers for the chromosome and plasmid are CP021454 and CP021871, respectively.

In Silico Analysis

For genome assembly of Illumina reads we used Spades V.3.6 (*3*). Annotation was performed with Prokka V1.11 (*4*) using standard default parameter settings. PacBio data were assembled de novo based on 59,447 PacBio long reads with an average read length of 10,355 bp using RS_HGAP_Assembly.3, included in the SMRT Portal version 2.3.0. Illumina short reads were mapped onto the assembled sequences using Burrows–Wheeler Aligner to obtain a highly accurate genome with QV60 final quality. Assembly quality was assessed through QUAST v2.3 (*5*), and contigs with >500 bp were considered for further analysis. Multilocus sequence typing (MLST) was carried out by mlst-package (https://github.com/tseemann/mlst). The *bla*$_{CTX-M}$ profiles, fim-type, serotype, and virulence gene were determined by Resfinder, FimTyper,

SeroTypeFinder, and VirulenceFinder, respectively (*6*–*9*). Plasmid incompatibility groups and plasmid MLST was performed using PlasmidFinder and pMLST (*10*). The presence of the M27PP1 region was determined by LS-BSR (*11*). For core genome analysis, draft genomes of the 24 isolates from Germany were compared with isolates from Japan (n = 13) using Harvest Suite (version 1.2) (*12*) with a default parameter *E. coli* EC958 and *E. coli* H105 were used as reference genomes for between- and within-clade comparisons (Technical Appendix Figure). Genome sequencing data for the *bla*$_{CTX-M-27}$-encoding isolates are deposited in European Nucleotide Archive under accession no. PRJEB21697.

References

1. Falgenhauer L, Waezsada S-E, Gwozdzinski K, Ghosh H, Doijad S, Bunk B, et al. Chromosomal Locations of *mcr-1* and *bla* $_{CTX-M-15}$ in Fluoroquinolone-Resistant *Escherichia coli* ST410. Emerg Infect Dis. 2016;22:1689–91. PubMed http://dx.doi.org/10.3201/eid2209.160692

2. Fritzenwanker M, Imirzalioglu C, Gentil K, Falgenhauer L, Wagenlehner FM, Chakraborty T. Incidental detection of a urinary *Escherichia coli* isolate harbouring *mcr-1* of a patient with no history of colistin treatment. Clin Microbiol Infect. 2016;22:954–5. PubMed http://dx.doi.org/10.1016/j.cmi.2016.08.027

3. Bankevich A, Nurk S, Antipov D, Gurevich AA, Dvorkin M, Kulikov AS, et al. SPAdes: a new genome assembly algorithm and its applications to single-cell sequencing. J Comput Biol. 2012;19:455–77. PubMed http://dx.doi.org/10.1089/cmb.2012.0021

4. Seemann T. Prokka: rapid prokaryotic genome annotation. Bioinformatics. 2014;30:2068–9. PubMed http://dx.doi.org/10.1093/bioinformatics/btu153

5. Gurevich A, Saveliev V, Vyahhi N, Tesler G. QUAST: quality assessment tool for genome assemblies. Bioinformatics. 2013;29:1072–5. PubMed http://dx.doi.org/10.1093/bioinformatics/btt086

6. Zankari E, Hasman H, Cosentino S, Vestergaard M, Rasmussen S, Lund O, et al. Identification of acquired antimicrobial resistance genes. J Antimicrob Chemother. 2012;67:2640–4. PubMed http://dx.doi.org/10.1093/jac/dks261

7. Roer L, Tchesnokova V, Allesøe R, Muradova M, Chattopadhyay S, Ahrenfeldt J, et al. Development of a Web tool for *Escherichia coli* subtyping based on fimH alleles. J Clin Microbiol. 2017;55:2538–43. PubMed http://dx.doi.org/10.1128/JCM.00737-17

8. Joensen KG, Tetzschner AMM, Iguchi A, Aarestrup FM, Scheutz F. Rapid and easy in silico serotyping of *Escherichia coli* isolates by use of whole-genome sequencing data. J Clin Microbiol. 2015;53:2410–26. PubMed http://dx.doi.org/10.1128/JCM.00008-15

9. Joensen KG, Scheutz F, Lund O, Hasman H, Kaas RS, Nielsen EM, et al. Real-time whole-genome sequencing for routine typing, surveillance, and outbreak detection of verotoxigenic *Escherichia coli*. J Clin Microbiol. 2014;52:1501–10. PubMed http://dx.doi.org/10.1128/JCM.03617-13

10. Carattoli A, Zankari E, García-Fernández A, Voldby Larsen M, Lund O, Villa L, et al. In silico detection and typing of plasmids using PlasmidFinder and plasmid multilocus sequence typing. Antimicrob Agents Chemother. 2014;58:3895–903. PubMed http://dx.doi.org/10.1128/AAC.02412-14

11. Sahl JW, Caporaso JG, Rasko DA, Keim P. The large-scale blast score ratio (LS-BSR) pipeline: a method to rapidly compare genetic content between bacterial genomes. PeerJ. 2014;2:e332. PubMed http://dx.doi.org/10.7717/peerj.332

12. Treangen TJ, Ondov BD, Koren S, Phillippy AM. The Harvest suite for rapid core-genome alignment and visualization of thousands of intraspecific microbial genomes. Genome Biol. 2014;15:524. PubMed http://dx.doi.org/10.1186/s13059-014-0524-x

13. Matsumura Y, Pitout JDD, Gomi R, Matsuda T, Noguchi T, Yamamoto M, et al. Global *Escherichia coli* sequence type 131 clade with $bla_{CTX\text{-}M\text{-}27}$ gene. Emerg Infect Dis. 2016;22:1900–7. PubMed http://dx.doi.org/10.3201/eid2211.160519

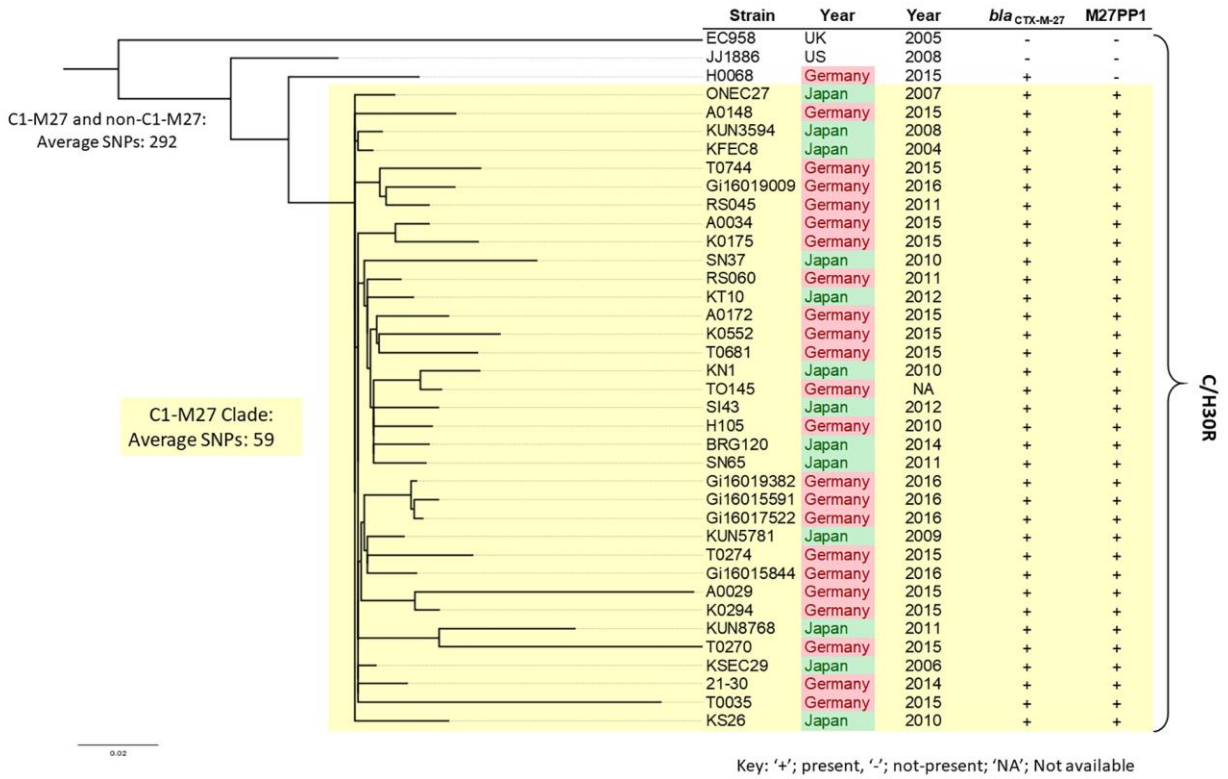

Technical Appendix Figure. Core genome based phylogenomic analysis of *Escherichia coli* sequence type (ST) 131 C1-M27 isolates from Germany and Japan (*13*). The tree is rooted to EC958. An average of 59 single-nucleotide polymorphisms were identified in core genome of C1-M27 clade, whereas an average of 292 single-nucleotide polymorphisms was recognized between C1-M27 and non–C1-M27 clade. Scale bar indicates nucleotide substitutions per site.
